# Early penile metastasis from primary bladder cancer as the first systemic manifestation: a case report

**DOI:** 10.4076/1757-1626-2-7281

**Published:** 2009-08-14

**Authors:** Nurettin Cem Sönmez, Burhan Coşkun, Serdar Arisan, Soner Güney, Ayhan Dalkılıç

**Affiliations:** 1 Department of 1st Urology, Sisli Etfal Research Hospital, Şişli, Istanbul, 34360, Turkey

## Abstract

Metastatic involement of penis is an exceptionally rare condition. 77% of the metastases are originated from the pelvic region; prostate and bladder are the most frequent primary locations. Retrograde venous route, retrograde lymphatic route, arterial spread, direct extension, implantation and secondary to instrumentation are the mechanisms of metastasis. Approximately two thirds of all penile metastasis are detected at a mean time of 18 months after the detection of the primary tumor and the remaining one third is presented at the same time with primary tumor. Diagnosis is usually made by biopsy and also non invasive methods as MRI or colour-coded duplex ultrasonography. Treatment options in these patients are local excision, partial or complete penectomy, external beam radiation therapy and chemotheraphy. Despite these alternatives prognosis is usually poor.

We present a case of urethelial carcinoma of the bladder and coincidental prostate adenocarcinoma with penile metastasis which is presented with priapism 6 months after radical cystectomy as the first systemic manifestation. We performed biopsy initially for staging and the patient underwent MRI showing the extension of the disease. The patient underwent radiotherapy of 56 gy and priapism partially resolved after the treatment. Chemotheraphy was also planned but the patient died 3 months following radiotheraphy.

## Introduction

Metastatic involement of penis is an exceptionally rare condition. The first penile metastasis case was reported by Eberth in 1870 [1]. Approximately 370 more cases were reported up to 136 years period. Seventy seven percent of the metastasis are originated from pelvic region and prostate and bladder is the most frequent primary location [2]. We present a case of urethelial carcinoma of bladder and coincidental prostate adenocarcinoma with penile metastasis which is presented with priapism 6 months after radical cystectomy as the first systemic manifestation.

## Case presentation

**Figure 1:**
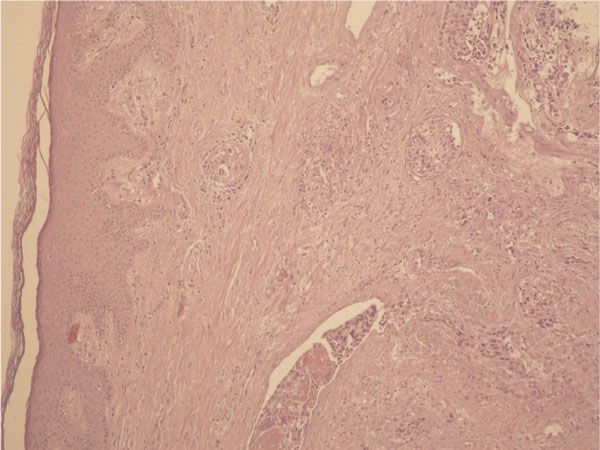
**Tumoral infiltration of glans penis**.

**Figure 2:**
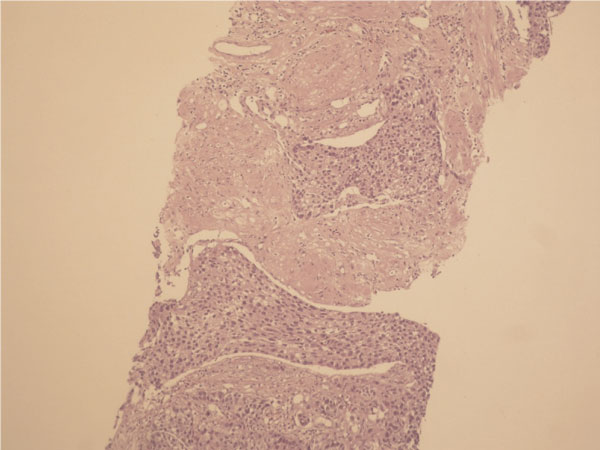
**Tumoral infiltration of corpus cavernosum**.

**Figure 3:**
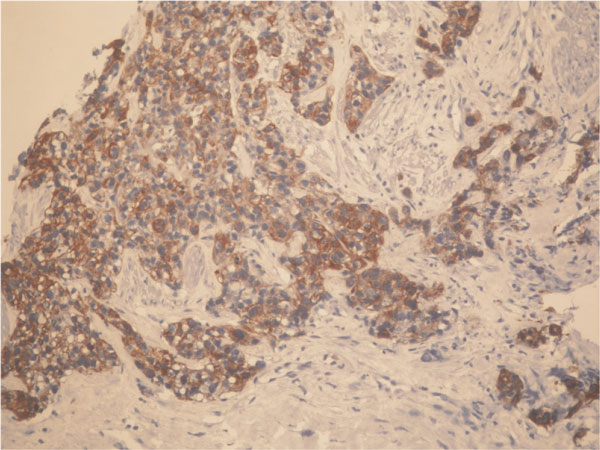
**Immunohistochemical staining of tumor cells**.

**Figure 4:**
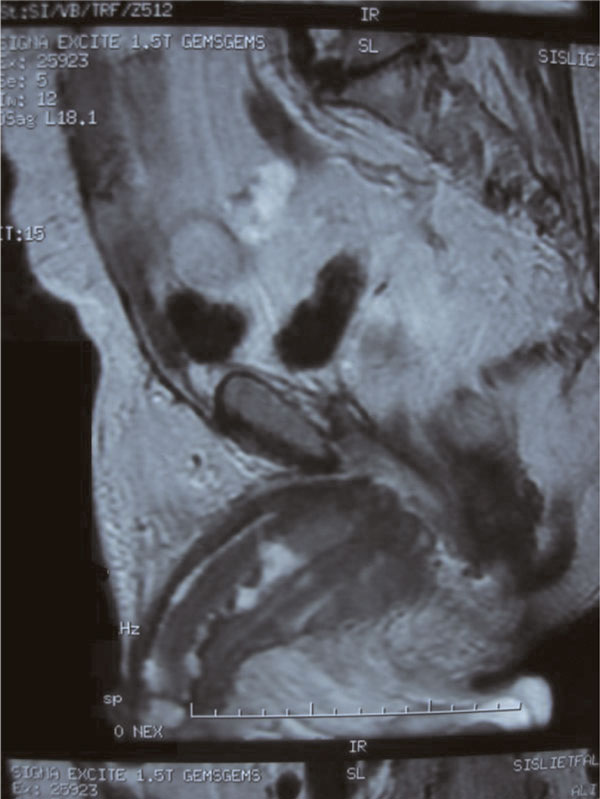
**Infiltration of cavernosal bodies on MRI**.

A 74-year-old Turkish Caucasian male patient, with high grade urethelial carcinoma of the bladder with evidence of lamina propria invasion was referred in January 2007. He had the diagnosis 9 months before that time, and after the operation 6 cycles of cisplatin and gemcitabine combination was given to patient by a medical oncology clinic from an outside center. He had smoked for 20 years and had intermittant painless hematuria for 2 months before the initial diagnosis. Digital rectal examination was unremarkable and the physical examination revealed normal. Laboratory data revealed hematocrit 40.2%, hemoglobin 12.8 ng/dl Urea: 54 mg/dl, creatinin: 1.21%/mg and PSA: 3.8 ng/dl. On cystoscopy, a broad based papillary tumor about 3 cm at the dome was diagnosed. Histological examination of the biopsy specimen revealed high-grade urethelial carcinoma of the bladder with invasion of the muscularis propria. He underwent radical cystoprostatectomy and bilateral pelvic node disection with an ileal conduit diversion in February 2007. Pathological examinaton revealed high-grade, infiltrative urethelial carcinoma of the bladder (pT3a/WHO 2004) surgical margins were negative and carcinoma insitu was not detected. Incidentally, prostatic adenocarcinoma, Gleason's score of 8 (4 + 4) with invasion at the apical surgical margin and left seminal vesicle was detected. External iliac, obturator lymph nodes and distal end biopsies of both ureters were negative for neoplasia. Six months after cystectomy, patient presented with priapism, pain, and urethral discharge. On physical examination penis was erect and swollen, nodules were palpated at glans, left and right side of the penile shaft. On digital rectal examination there was no suspicion for tumor involvement. PSA level of the patient was 0.002 ng/dl. An excisional biopsy from glans and tru-cut biopsies from cavernosal bodies were performed. Pathological examination of the excisional biopsy revealed, infiltration of carcinoma at subephitelial area with vascular invasion (Figure 1) and the tru-cut biopsies also revealed carcinoma infiltration (Figure 2). According to immunohistochemical studies tumor cells were broadly stained positive for cytokeratin 7, high molecular weighted cytokeratin, focally positive for cytokeratin 20 and negative for PSA (Figure 3). Morphological and immunohistochemical findings were concordant with infiltration of urethelial carcinoma. Metastatic work up also revealed chest metastasis. Pelvic MRI was also used for staging the patient and showed progression with multiple nodules which infiltrate cavernosal bodies (Figure 4). Patient underwent radiotherapy of 56 gy and priapism partially resolved after the treatment. Chemoteraphy was also planned for the patient due to chest metastasis but the patient died 3 months following radiotheraphy.

## Discussion

It is still unknown, why does penile metastasis is infrequent despite the fact that penis has got rich vascularazition and intensive venous communication with neighbouring organs. Up to date over 370 cases are reported [2]. Retrograde venous route, retrograde lymphatic route, arterial spread, direct extension, implantation and secondary to instrumentation are the mechanisims of metastase as described by Paquin and Roland [3]. In a review of 372 penile metastasis cases presented by Cherian et al. in 2006, primary tumor localization was prostate, bladder, recto-sigmoid and rectum, and kidney in 34, 30, 13, 8 percent of the cases respectively [2]. Approximately-two thirds of all penile metastasis are detected at a mean time of 18 months after the detection of the primary tumor and remaing one third is presented at the same time with primary tumor [4]. Although most of the penile metastase developed at the same time with other metastases, there are cases of late penile involvement with long survey after adequate treatment [5]. In our case, penile metastasis developed 6 months after radical cystectomy unusually as the first manifestation of the systemic disease. The fact that patient had a deffered definitive treatment of bladder cancer may play a role in development of early metastase but penile region is unexpected. Mass, induration and nodules are the initial presentation of penile metastasis in 51%, priapism in 27%, urinary symptoms like hemorrage, hematuria, incontinence, and irritative and obstructive symptoms in 27%, pain in 17%, retention in 13% and skin lesions in 11% of the patients [6]. Mechanism of metastase can be explained by obstruction or thrombosis of the corpora cavernosa or irritation of the neural pathways caused by metastatic tumor [7]. Our patient presented with priapism, nodules and pain suggesting the progression of the disease. Diagnosis is usually made by biopsy and also non invasive methods as MRI or colour-coded duplex ultrasonography may be choosen for staging the disease [2-8]. We performed biopsy initially for staging, the patient underwent MRI and images showed the extension of disease. Treatment options in these patients are local excision, partial or complete penectomy, external beam radiation therapy and chemotheraphy. Despite this alternatives prognosis is usually poor [2]. Most of the patients with penile metastasis have advanced disease and survival after the diagnosis is generally short and 80% of the patients are lost within 6 months [9].

## Abbreviations

Gy: gray; MRI: magnetic resonance imaging; PSA: prostate specific antigen.

## Consent

Written informed consent was obtained from the patient's son for publication of this case report and accompanying images. A copy of the written consent is available for review by the Editor-in-Chief of this journal.

## Competing interests

The authors declare that they have no competing interests.

## Authors' contributions

NCS was the organizer of the case report, surgeon responsible for the diagnosis. BC was the organizer and resident responsible for the follow up of the case. SA was the surgeon responsible for the radical cystectomy and researcher of the literature. SG was the surgeon responsible for the radical cystectomy and researcher of the literature. AD was the authority and director of the case report.
